# Hydrothermal Preparation and White-Light-Controlled Resistive Switching Behavior of BaWO_4_ Nanospheres

**DOI:** 10.1007/s40820-014-0021-5

**Published:** 2014-11-22

**Authors:** Bai Sun, Yonghong Liu, Wenxi Zhao, Jinggao Wu, Peng Chen

**Affiliations:** 1 School of Physics Science and Technology, Southwest University, Chongqing, 400715 People’s Republic of China; 2 Institute for Clean Energy & Advanced Materials (ICEAM), Southwest University, Chongqing, 400715 People’s Republic of China

**Keywords:** BaWO_4_ nanospheres, Resistive switching, Hydrothermal preparation, White light

## Abstract

In this work, BaWO_4_ nanospheres were successfully prepared by hydrothermal process. The bipolar resistive switching behavior of Ag/BaWO_4_/FTO device is observed. Moreover, this resistive switching behavior can be modulated by white light. The device can maintain superior stability in the dark and under white-light illumination. This study is useful for developing the light-controlled nonvolatile memory devices.

## Introduction

Reversible resistive-switching effect is a promising candidate for next-generation nonvolatile memories [1]. The resistive switching behavior, in which the reversible switching between a high-resistance state (HRS) and a low-resistance state (LRS) can be achieved by the applied voltage, is an attractive subject of scientific and technical research [2–6]. The resistive switching is classified into unipolar resistive switching and bipolar resistive switching [7]. The resistive switching memory cell usually has simple structure, in which an insulating oxide is sandwiched between two metal electrodes [8]. Therefore, the resistive switching device is suitable for wide application because of the simple preparation steps and relatively low cost.

In the past few years, a new control method (light controlled) has been involved in the resistive switching memory device. Ungureanu firstly reported the light-controlled resistive switching memory in Pd/Al_2_O_3_/SiO_2_ device [9]. At the same time, Adachi and Park also added the light as extra control parameter in the switching memory device based on ZnO nanorods [10–12]. In addition, our group also found that light can act as a control method in some resistive switching systems [13–15]. The light-controlled resistive switching effect provides the potential for light-controlled nonvolatile memory device, which may be a promising developing trend of information science and storage technology. In addition, the white light, which is the most ordinary light source, is widely used.

BaWO_4_ is a wide gap semiconductor with Eg >4.9 eV and has a Scheelite structure [16, 17]. BaWO_4_ is an important material in the electro-optical industry owing to its emission of blue luminescence [18–23]. Therefore, BaWO_4_ received more and more research interest [24].

Although there are many reports about various applications in BaWO_4_ nanostructure in previous works, the resistive switching properties of BaWO_4_ have not been reported yet. Herein we present the reversible bipolar resistive-switching effect in Ag/BaWO_4_/FTO device. Moreover, the resistive-switching effect can be controlled by white-light illumination.

## Experimental

### Preparation of BaWO_4_ Nanospheres

The BaWO_4_ nanospheres were prepared by a hydrothermal process using cetyltrimethylammonium bromide (CTAB) as the surfactant. All the chemicals used in this work were of analytical grade and used directly without further purification. The distilled water was used as a solvent throughout the experiment. Firstly, Ba(NO_3_)_2_ (0.01 M) and Na_2_WO_4_·2H_2_O (0.01 M) were dissolved in 40 ml distilled water under stirring continuously. Then 0.5 g cationic surfactant cetyltrimethylammonium bromide (CTAB) was added into above solution under strong stirring. After continuous stirring for 2 h, the solution was transferred to a 50-ml sealed Teflon-lined steel autoclave. Then, the sealed Teflon-lined steel autoclave was heated and kept at 200 °C for 72 h. After the autoclave was cooled to room temperature, the powder obtained was washed with distilled water and ethanol and dried at 60 °C for 12 h.

### Preparation of Ag/BaWO_4_/FTO Device

Firstly, FTO substrates were cleaned by acetone, ethanol, and deionized water, and subsequently dried on the spin coater. Secondly, BaWO_4_ films were prepared on FTO substrate by spin-coating method. The detail preparation process of BaWO_4_ films is as follows: Firstly, we grinded the as-prepared BaWO_4_ nanospheres powder for 2 h. Next, we dissolved the powder in toluene solution to prepare precursor gel. Then the precursor gel was spin-coated on the FTO substrate. The spin-coating process at 5,000 rpm for 10 s was used to prepare BaWO_4_ films with thickness of about 2 μm. Then these samples were subsequently dried at 60 °C in vacuum for overnight. The thickness of the BaWO_4_ film was detected by the step profiler.

## Characterizations

**Fig. 1:**
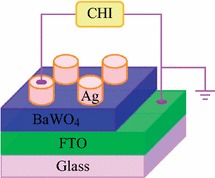
The schematic representation of *I*–*V* measurement

Crystal structure of BaWO_4_ nanospheres was characterized by X-ray diffraction (XRD) with Cu *Kα* radiation at room temperature. Surface morphology of BaWO_4_ nanospheres was characterized using scanning electron microscope (SEM). Microstructure, nanosphere size, selected area electron diffraction (SAED) pattern, and the energy-dispersive X-ray spectroscopy (EDX) spectra of the BaWO_4_ nanospheres were observed by transmission electron microscopy (TEM) at an acceleration voltage of 200 kV. In the test of resistive switching characterizations, Ag is top electrode and FTO is bottom electrode, as shown in Fig. 1. Ag electrodes with area of ~1 mm^2^ and thickness of 200 nm were prepared by vacuum deposition. And the preparation process of Ag electrodes is as follows: Firstly, we covered a mask on surface of BaWO_4_/FTO. Secondly, we put it into the vacuum sputtering system to grow Ag electrodes. Finally, we chose the superior electrodes for characterization. Current–voltage (*I*–*V*) and resistance cycles curves were tested using the electrochemical workstation (CHI) at room temperature. In addition, we used an ordinary filament lamp as light source. The wavelength range of light is 400–760 nm.

## Results and Discussion

Figure 1 shows the schematic representation of the device for *I*–*V* measurement, where the BaWO_4_ film with thickness of ~2 μm was spin coated on the FTO substrate, and the electrodes of Ag with the area of less than 1 mm^2^ and thickness of 200 nm were deposited onto the BaWO_4_ film.

**Fig. 2:**
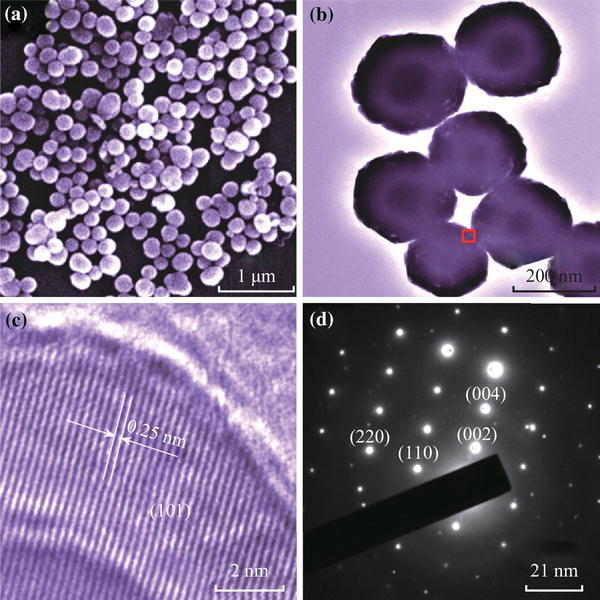
**a** The SEM image of the as-prepared BaWO_4_ nanospheres. **b** The TEM image of BaWO_4_ nanospheres. **c** The HRTEM of a typical portion recorded in the *rectangular area* of part (**b**). **d** The SAED pattern of BaWO_4_ nanospheres

Scanning electron microscope (SEM) image of the as-prepared BaWO_4_ nanospheres is shown in Fig. 2a. The as-prepared sample consists of BaWO_4_ nanospheres. And the size of these nanospheres is about 180–220 nm from the transmission electron microscopy (TEM) image in Fig. 2b. From the high-resolution transmission electron microscopy (HRTEM) image of BaWO_4_ nanospheres in Fig. 2c, the lattice spacing between two planes is ~0.25 nm, corresponding to the (101) planes of BaWO_4_. Figure 2d exhibits the selected area electron diffraction (SAED) pattern of the BaWO_4_ nanospheres, where the corresponding nearest four spots in the figure can be indexed to (110), (220), (002), and (004) planes of BaWO_4_, indicating that as-prepared BaWO_4_ nanospheres possess an excellent single-crystal structure.

**Fig. 3:**
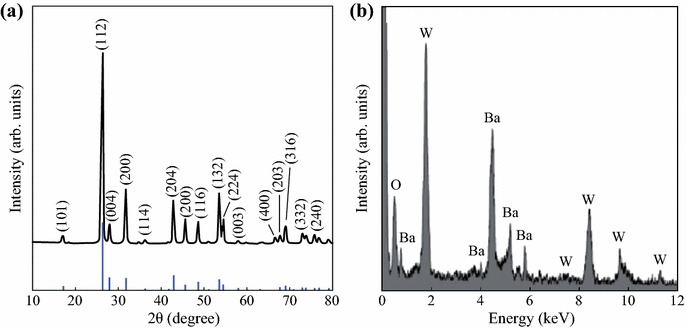
**a** The XRD of as-prepared BaWO_4_ nanospheres at room temperature. **b** The EDX spectrum of BaWO_4_ nanospheres

The crystalline structure of the BaWO_4_ nanospheres was characterized by XRD. Figure 3a exhibits the XRD pattern of as-prepared BaWO_4_ nanospheres. There are only the peaks of BaWO_4_, which reveals the purity of the BaWO_4_ nanospheres. The XRD demonstrates the characteristic diffraction peaks of BaWO_4_. Moreover, the XRD profile matches very well with that in the reported work [25–28]. The result indicates that the BaWO_4_ nanospheres have a tetragonal scheelite unit cell (*a* = 5.62 ± 0.05 Å, *c* = 12.71 ± 0.07 Å) according to the peak positions and their relative intensities, which is consistent with the reported value (JCPDS Cards 08-457). Therefore, the product contains only pure BaWO_4_, and the sharp peaks demonstrate good crystallinity of the BaWO_4_ nanospheres. The composition of BaWO_4_ nanospheres was further confirmed by elemental analysis carried out with energy-dispersive X-ray spectra (EDX). The EDX data in Fig. 3b confirm that the compositions of as-prepared product are only Ba, W, and O with an atomic ratio of 0.93:0.98:4, which is close to the stoichiometric ratio of BaWO_4_.

**Fig. 4:**
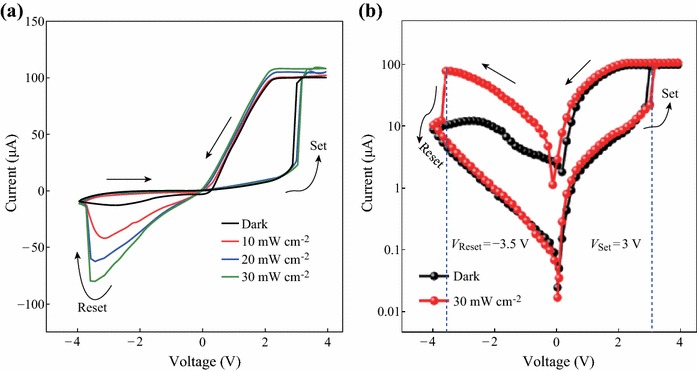
**a** The *I*–*V* characteristic curves in linear scale of Ag/BaWO_4_/FTO structure in the dark and under white-light illumination with power density of 30 mW cm^−2^. **b** The corresponding *I*–*V* characteristic curves in logarithmic scale

Figure 4a displays the *I*–*V* characteristics curves of Ag/BaWO_4_/FTO device in linear scale in the dark and under white-light illumination with power density of 30 mW cm^−2^, we can see that *I*–*V* curves exhibit asymmetric behavior with significant hysteresis. The arrows in the figure denote the sweeping direction of voltage.

Figure 4b presents a corresponding *I*–*V* curve of Ag/BaWO_4_/FTO device in logarithmic scale. The arrows in the figure denote the sweeping direction of voltage. The Ag/BaWO_4_/FTO device shows obvious resistive switching behavior in the dark. A sudden current increasing occurs at 3.0 V (*V*_Set_), indicating a resistive switching from the high-resistance state (HRS or ‘OFF’) to the low-resistance state (LRS or ‘ON’), which was called the “Set” process. When the applied voltage sweeps from zero to negative voltage of about −3.5 V (*V*_Reset_), the device can return to the HRS, which was called the “Reset” process. The resistances of HRS and LRS at negative bias are much larger than those at positive bias. During the successive “Set” and “Reset” cycles on the same device, the device shows the identical *I*–*V* curves. The *V*_Reset_ and *V*_Set_ are almost unchanged in subsequent cycles for the same device (not shown here). Moreover, the resistive switching behavior of Ag/BaWO_4_/FTO device is improved by white-light illumination. The *I*–*V* curve under white-light illumination is more symmetrical than that in the dark. And the resistive switching behavior at negative bias is more obvious than that in the dark. Furthermore, the resistance of LRS at negative bias is nearly as same as that at positive bias. In addition, the *V*_Set_ (3.1 V) under white-light illumination is larger than that (3.0 V) in the dark.

**Fig. 5:**
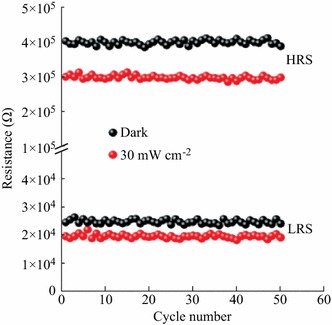
The resistance cycles curve with a positive bias voltage of 1.0 V in the dark and under white-light illumination with power density of 30 mW cm^−2^

In order to estimate the probable practicability of the white-light-controlled resistive switching behaviors of the Ag/BaWO_4_/FTO device, the resistance cycles number curves for the HRS and LRS with a positive bias of 1.0 V in the dark and under illumination with power density of 30 mW cm^−2^ are tested and shown in Fig. 5. The resistances are about 25 kΩ at the LRS (ON state) and 400 kΩ at the HRS (OFF state) in the dark, indicating the OFF/ON-state resistance ratio is up to 16. However, the resistances are about 20 kΩ at the LRS (ON state) and 300 kΩ at the HRS (OFF state) under white-light illumination, suggesting the OFF/ON-state resistance ratio is 15. More importantly, the resistances of the LRS (ON state) and the HRS (OFF state) are nearly unchanged after 50 cycles for the device in the dark and under white-light illumination, which indicates the good stability of the white-light-controlled resistive switching behaviors of the Ag/BaWO_4_/FTO device. According to the above results, the steady white-light-controlled resistive switching behavior in Ag/BaWO_4_/FTO structure provides the potential for light-controlled nonvolatile optoelectronic memory applications.

The mechanism for resistive switching in a metal/oxides/oxides structure has been extensively investigated [8, 29–33]. In our works, current–voltage curve of the Ag/BaWO_4_/Ag structure is symmetrically linear without hysteresis (not shown here), indicating it is Ohmic contact between Ag and BaWO_4_. Therefore, the asymmetric behavior of *I*–*V* curve of Ag/BaWO_4_/FTO in the dark indicates that a Schottky barrier is formed at the interface of BaWO_4_/FTO. The bipolar resistive switching behavior of Ag/BaWO_4_/FTO should result from the trapped and detrapped charge in the Schottky-like depletion layer [26–31]. Moreover, the white light can generate a large number of charges, which can change the trapped state and detrapped state in the Schottky-like depletion layer [9–12]. Therefore, the white light can modulate the resistive switching behavior of Ag/BaWO_4_/FTO.

## Conclusions

BaWO_4_ nanospheres were prepared by hydrothermal process. The reversible bipolar resistive switching characteristics of Ag/BaWO_4_/FTO device were observed. In particularly, the resistance switching behavior can be controlled by white-light illumination. Therefore, the superior resistance switching characteristics of the Ag/BaWO_4_/FTO device hold a promise for light-controlled nonvolatile memory applications.
